# Clinical significance of HER2 and EGFR expression in colorectal cancer patients with ovarian metastasis

**DOI:** 10.1186/s12907-019-0085-8

**Published:** 2019-02-28

**Authors:** Ji-Lin Li, Shu-Han Lin, Hong-Qiu Chen, Li-Sheng Liang, Xian-Wei Mo, Hao Lai, Jie Zhang, Jing Xu, Bing-Qian Gao, Yan Feng, Yuan Lin

**Affiliations:** 1 Gastrointestinal Surgery Department, Affiliated Cancer Hospital of Guangxi Medical University, 71 Hedi Road, Nanning, Guangxi Zhuang Autonomous Region 530021 People’s Republic of China; 2 Hepatobiliary surgery, People’s Hospital of Guigang, 1 Jianshe Road, Guigang, Guangxi Zhuang Autonomous Region 537100 People’s Republic of China; 3 Department of Pathology, People’s Hospital of Guigang, 1 Jianshe Road, Guigang, Guangxi Zhuang Autonomous Region 537100 People’s Republic of China; 4 Research Department, Affiliated Cancer Hospital of Guangxi Medical University, 71 Hedi Road, Nanning, Guangxi Zhuang Autonomous Region 530021 People’s Republic of China

**Keywords:** Colorectal cancer, Ovarian metastases, HER2, EGFR, Prognosis

## Abstract

**Background:**

EGFR and HER2 overexpression has been reported to play important roles in colorectal cancer (CRC) development and metastasis. Ovarian metastasis is rare yet is one of the most malignant metastases of CRC, but very few studies have focused on its biological features. This study aimed to investigate the expression of EGFR and HER2 in ovarian metastases of CRC and to reveal their clinical significance.

**Methods:**

The expression of HER2 and EGFR in both primary tumours and ovarian metastases was analysed by immunohistochemistry (IHC) in 31 CRC patients with ovarian metastases as well as in the primary tumours of 26 CRC patients with non-ovarian metastases. The overall survival time was calculated with a Kaplan-Meier survival curve and compared with a log-rank test.

**Results:**

HER2 positivity in primary tumours was significantly higher in patients with ovarian metastases than in those with non-ovarian metastases (54.5% vs. 36.4%, *P* < 0.05). The EGFR-positive rate in primary lesions was not significantly different between patients with ovarian metastases and those with non-ovarian metastases (63.6% vs. 58.3%, *P* > 0.05). HER2 expression was not correlated with age, primary tumour site, tumour differentiation, tumour diameter or vascular cancer embolus (*P* > 0.05). The positive rates of HER2 and EGFR in ovarian metastases were 44.8 and 69.0%, respectively. HER2 expression in ovarian metastases was correlated with peritoneal metastasis and bilateral ovarian metastasis (*P* < 0.05) but not with age, synchronous or metachronous ovarian metastases and the primary tumour site (*P* > 0.05). There was no significant correlation between EGFR expression and the clinicopathological features in ovarian metastases (*P* > 0.05). CRC patients with HER2-positive ovarian metastases showed a shortened overall survival time compared to that of CRC patients with HER2-negative metastases (17.0 ± 5.2 vs. 32.0 ± 8.3 months).

**Conclusion:**

Our studies revealed that EGFR and HER2 are highly expressed in the primary tumours and metastases of CRC patients with ovarian metastases. HER2 positivity may be a negative prognostic predictor in patients with ovarian metastases.

## Background

Colorectal cancer (CRC) is one of the most prevalent cancers worldwide, with 1.01 million new cases and > 0.55 million deaths/year [1]. Although the diagnosis and treatment of CRC has made great progress in recent years, the 5-year survival rate of patients can reach 90.3%, but the survival rate declines to 70.4 and 12.5% for patients diagnosed with regional and metastatic disease, respectively [2]. After the liver, the ovary is the most common metastatic organ in women with CRC [3]. Although the incidence of ovarian metastases is low, women with ovarian metastases have rapid disease progression and a poor prognosis [4, 5]. The overall median survival time of ovarian metastases reported is only 13.6 months [6]; therefore, understanding the mechanism of ovarian metastasis of CRC is of great significance for the prevention and treatment of ovarian metastases.

The human epidermal growth factor receptor family consists of four members: EGFR (HER1, erbB1), HER2 (erbB2, neu), HER3 (erbB3) and HER4 (erbB4), all of which regulate the proliferation and differentiation of various tumour cells [7]. EGFR is positively expressed in 59 to 85% of CRC specimens, and its overexpression is closely related to clinical stage, lymph node metastasis, disease-free survival, poor overall survival, and 5-year recurrence rate [8–10]. HER2 is an emerging therapeutic target and prognostic factor for metastatic CRC [11]. The positive expression rate of HER2 protein in CRC tumours varied from 2 to 11%, and this rate increased in more advanced diseases [12].

The expression of HER2 and EGFR and the expression of ovarian metastases in CRC have not been reported. This study compared the expression of HER2 and EGFR in ovarian and non-ovarian metastases in advanced CRC patients and assessed the relationship of their expression levels with clinicopathological variables. We further compared the expression of HER2 and EGFR in primary tumours and ovarian metastases in CRC patients with ovarian metastases and their influence on prognosis.

## Methods

### Patient information

A total of 31cases of CRC ovarian metastases from the Department of Gastrointestinal Surgery, Guangxi Medical University Affiliated Cancer Hospital from June 2013 to December 2017 were selected. The average age was 50.0 ± 11.0 years. There were 14 cases of synchronous metastases, 17 cases of metachronous metastases, 12 cases of unilateral ovarian metastases, and 19 cases of bilateral metastases; 20 cases had paired primary lesions and ovarian metastases, 9 cases had ovarian metastases only, and 2 cases had primary tumours only. The inclusion criteria were as follows: (1) postoperative diagnosis of primary CRC based on histopathology; and (2) ovarian metastases confirmed by postoperative pathology. Besides histological analysis, expressions of CK7, CA125, CK20, CEA, CDX2, PAX8 were also determined by immunohistochemistry to confirm ovarian metastasis when necessary. The exclusion criterion was the presence of primary ovarian cancer or ovarian metastases derived from other cancers. At the same time, retrospective analysis of third-stage patients with non-ovarian metastases who were treated at our hospital as the control group were selected as controls for EGFR or HER2. The inclusion criteria for the controls were (1) female patients with primary colorectal tumours confirmed by postoperative histopathology; (2) no ovarian metastases detected by computed tomography; and (3) previous confirmation of the HER2 or EGFR expression status with immunohistochemistry (IHC). In total, 22 patients were included in the HER2 control group, with an average age of 65.4 ± 14.5 years; four cases had liver metastases, one case had bladder metastasis, two cases had peritoneal metastasis, and 15 cases had tumour deposits in perirectal/mesorectal adipose tissues. The EGFR control group included 24 patients, with an average age of 67.6 ± 11.9 years, six cases had liver metastases, one had lung metastases, two had bone metastases (1 combined with lung metastases), one had bladder metastases, two had abdominal metastases, and 13 cases had tumour deposits in perirectal/mesorectal adipose tissues. The study was approved by the Ethics Committee of the Affiliated Cancer Hospital of Guangxi Medical University, and all patients signed informed consent.

### Immunohistochemical analysis

Surgical specimens were fixed in 10% neutral formaldehyde solution, dehydrated, embedded in paraffin, and serially sectioned at a thickness of 4 μm. Serial sections from the same blocks were used for HER2 and EGFR analysis. Specimen sections were dewaxed by xylene, rehydrated with a standard ethanol gradient, and subjected to high temperature and high pressure for antigen retrieval. The slices were incubated in H_2_O_2_ for 10 min at room temperature, subjected to dropwise addition of the corresponding primary antibody followed by incubation at 4 °C overnight, rinsed with phosphate buffered saline (PBS), and subjected to dropwise addition of secondary antibody. A DAB solution was added to visualize the antibody binding, after which the sections were rinsed with distilled water, counterstained with haematoxylin, dehydrated with an ethanol gradient, and fixed with xylene and gelatin. Mouse anti-human HER2/c-erB-2 monoclonal antibody (product number: ZM-0065, clone number: UMAB36) and mouse anti-human EGFR monoclonal antibody (product number: ZM-0093, clone number: UMAB95) were purchased from Beijing Zhongshan Jinqiao Company. A ready-to-use rapid IHC MaxVision™^2^ detection kit (cat. KIT-5920) was purchased from Fuzhou Maixin Co., Ltd. The experimental procedure was carried out according to the manufacturer’s instructions. The negative control slices were incubated with PBS instead of primary antibody.

### Scoring system

HER2 is mostly localized to the cell membrane, while EGFR is expressed in the cell membrane and cytoplasm. HER2 expression was scored as described in the VENTANA study [13]: (−), no tumour cell staining or less than 10% cell membrane staining; (+), > 10% cells had weak or almost undetectable cell membrane staining; (++), > 10% cells were weak to moderate cell membrane staining; and (+++), > 10% of cells have strong cell membrane staining. EGFR expression was determined according to the staining intensity and the number of positive cells. The staining intensity was scored as follows: no staining, 0 points; staining faintly visible, 1 point; weak-medium staining, 2 points; and strong staining, 3 points. The number of positively stained cells was scored as follows: ≤ 10% positive cells, 1 point; 11 to 50%, 2 points; 51 to 80%, 3 points, and ≥ 80%, 4 points. The total score was calculated by multiplying the staining score by the positive cell number score. The expression of EGFR was determined as follows: 0 to 2 points, (−); 3 to 5 points, (+); 6 to 8 points, (++); and 9 to 12 points, (+++). For both HER2 and EGFR, (−) and (+) were considered negative, whereas (++) and (+++) were considered positive. All the results were independently assessed by two highly experienced pathologists in a double-blinded manner and agreed upon by consensus.

### Follow-up

All patients in this group were followed up mainly by outpatient review and telephone. The recorded survival time was from the time of diagnosis of ovarian metastases to the time of death or the last follow-up (August 2018).

### Statistics

All data were statistically analysed using SPSS 22. Comparisons of EGFR and HER2 expression between groups were using the Wilcoxon rank sum test. Comparisons of data according to HER2 or EGFR expression were performed by Fisher’s exact probability method. The correlation of HER2 and EGFR expression was performed by Spearman’s ranked correlation coefficient. Survival analysis was established according to the Kaplan-Meier method. Comparison of survival times was carried out using log-rank test. A *P*-value less than 0.05 was considered statistically significant.

## Results

### HER2 expression and clinicopathological features in primary tumours

**Fig. 1:**
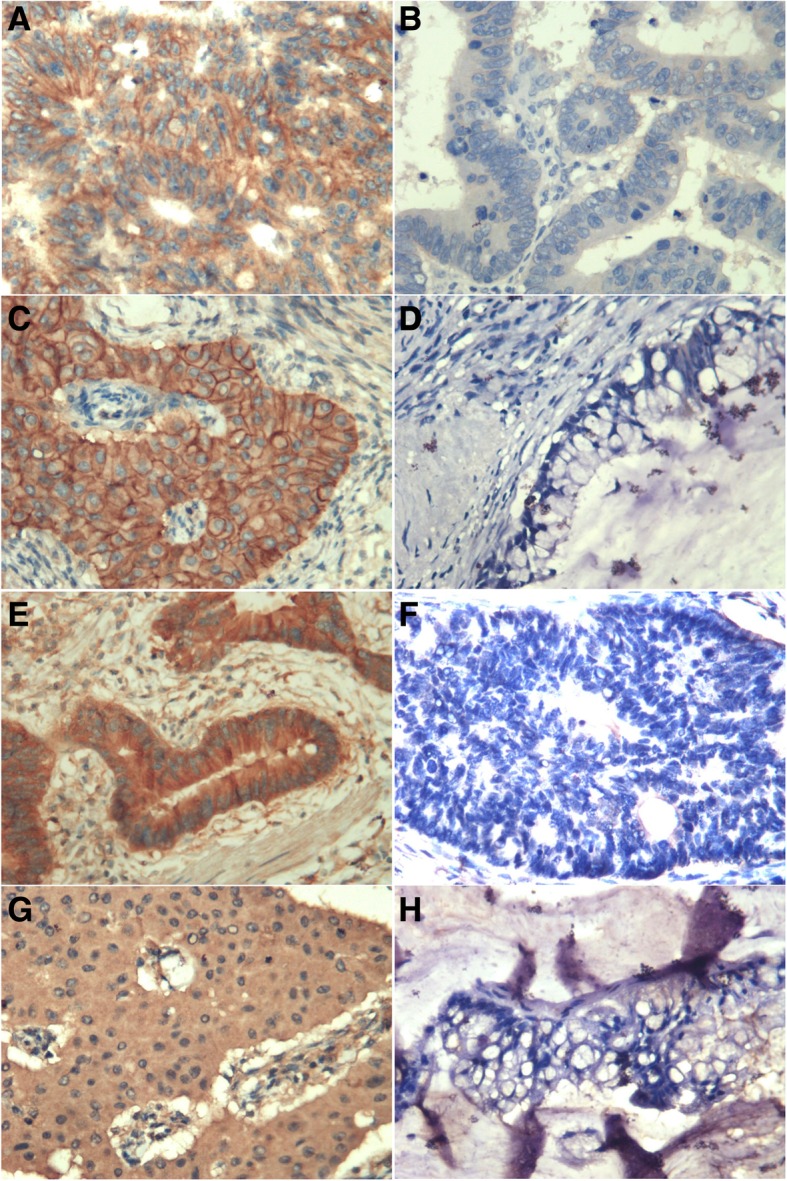
Assessment of protein expression of HER2 and EGFR by IHC staining. (× 400). **a, c,** Positive expression of HER2 in the primary lesion and ovarian metastases, respectively. **b, d,** Negative expression of HER2 in primary tumours and ovarian metastases, respectively. **e, g,** Positive expression of EGFR in primary lesion and ovarian metastases, respectively. **f, h,** Negative expression of EGFR in primary tumours and ovarian metastases, respectively

HER2 is mainly expressed in the cell membrane (Fig. 1 a-d). The primary tumour HER2-positive rate in ovarian metastatic CRC patients was 54.5%, which was significantly higher than that in HER2 control patients (36.4%, *P* < 0.05) (Table 1). There was no significant correlation in the expression of HER2 with age, tumour site, tumour differentiation, tumour diameter, number of positive mesenteric lymph nodes or vascular cancer embolus (*P* > 0.05). There was no significant correlation between the expression of HER2 and the clinicopathological features in CRC patients with non-ovarian metastasis (*P* > 0.05) (see Table 2).

**Table 1:** Expression of HER2 and EGFR in primary tumours of CRC patients with ovarian and non-ovarian metastases

		Total (*n*)	Expression (*n*)				Positive rate (%)	Z	*P*
			(−)	(+)	(++)	(+++)			
HER2	Ovarian metastasis	22	4	6	6	6	54.5	−2.004	0.045
	Non-ovarian metastasis	22	12	2	5	3	36.4		
EGFR	Ovarian metastasis	22	1	7	6	8	63.6	−1.055	0.315
	Non-ovarian metastasis	24	7	3	7	7	58.3

**Table 2:** Clinicopathological variables and their correlation with immunohistochemical expression of HER2 in primary tumours

Clinicopathological variables	Ovarian metastasis CRC patients				Non-Ovarian metastasis CRC patients		
	Total	Positive [n (%)]	*P′*		Total	Positive [n (%)]	*P″*
Total	22	12 (54.5)	***–***		22	8 (36.4)	***–***
Age							
≤ 55	15	9 (60.0)	0.652		8	1 (12.5)	0.167
> 55	7	3 (42.9)			14	7 (50.0)	
Primary tumour location							
Colon	17	9(52.9)	1.000		11	2 (18.2)	0.183
Rectum	5	3 (60.0)			11	6 (54.5)	
Differentiation							
High	2	2 (100)	0.476		1	1 (100)	0.364
Mid-Low	19	9 (47.4)			21	7 (33.3)	
unknown	1	1 (100)			0		
Positive lymph node							
0	5	4 (80.0)	0.150		8	2 (25.0)	0.853
1~3	6	5 (83.3)			9	4(44.4)	
≥ 4	9	3 (33.3)			5	2 (40.0)	
unknown	2	0 (0.0)					
Tumour diameters							
≤ 5 cm	11	7 (63.6)	0.653		15	5 (33.3)	1.000
> 5 cm	9	4 (44.4)			7	3 (42.9)	
unknown	2				0		
Vascular cancer embolus							
Yes	9	3 (33.3)	0.192		9	5 (55.6)	0.203
No	13	9 (69.2)			12	3 (25.0)	
unknown	0	0 (0.0)			2	0 (0.0)	

### EGFR expression and clinicopathological features in primary tumours

EGFR was localized to the cell membrane and cytoplasm (Fig. 1e-h). There was no significant difference in the expression of EGFR between CRC patients with ovarian metastasis and those with non-ovarian metastasis (*P* > 0.05, Table 2). The positive rate of EGFR expression in the CRC ovarian metastasis group was 63.6%. No significant difference was found between the expression of EGFR and the clinicopathological variables (*P* > 0.05, Table 3).

**Table 3:** Clinicopathological variables and their correlation with immunohistochemical expression of EGFR in primary tumours

Clinicopathological variables	CRC patients with ovarian metastasis			CRC patients with non-ovarian metastasis		
	Total	Positive [n (%)]	*P′*	Total	Positive [n (%)]	*P*″
Total	22	14 (63.6)		24	14 (58.3)	
Age						
≤ 55	15	9 (60)	1.000	6	3 (50.0)	0.665
> 55	7	5 (71.4)		18	11 (61.1)	
Primary tumour location						
Colon	17	10 (58.8)	0.613	14	8 (57.1)	1.000
Rectum	5	4 (80.0)		10	6 (60.0)	
Differentiation						
High	2	1 (50.0)	1.000	1	0 (0.0)	0.417
Mid-Low	19	12 (63.2)		23	14 (60.9)	
unknown	1	1 (100)				
Positive lymph node						
0	5	4 (80.0)	0.716	6	1 (16.7)	0.101
1~3	6	3 (50.0)		10	7 (70.0)	
≥ 4	9	6 (66.7)		8	6 (75.0)	
unknown	2	1 (50.0)		–	–	
Tumour diameters						
≤ 5 cm	11	8 (72.7)	0.642	16	9 (56.3)	1.000
> 5 cm	9	6 (66.7)		8	5 (62.5)	
unknown	2	1 (50)		–	–	
Vascular cancer embolus						
Yes	9	5 (55.6)	0.662	10	6 (60.0)	1.000
No	13	9 (69.2)		11	7 (63.6)	
unknown	0	0 (0.0)		3	1 (33.3)	

### Expression and clinicopathological features of HER2 and EGFR in ovarian metastases

The positive rate of HER2 in ovarian metastases was 44.8% (13/29). The expression of HER2 in ovarian metastases was associated with peritoneal metastasis and bilateral ovarian infiltration (*P* < 0.05, Table 4). HER2 expression in ovarian metastases showed no significant relationship with age, synchronous or metachronous ovarian metastasis, or primary site (P > 0.05, Table 4).

**Table 4:** Clinicopathological variables and their correlation with immunohistochemical expression of HER2 and EGFR in ovarian metastases

Clinical Variables	HER2 Expression				EGFR Expression			
	Total	Negative	Positive	*P*-value	Total	Negative	Positive	*P-*value
Total	29	16	13	**–**	29	9	20	**–**
Age								
≤ 55	19	10	9	1.000	19	6	13	1.000
> 55	10	6	4		10	3	7	
Ovarian metastasis								
Synchronous	13	5	8	0.144	13	4	9	1.000
Metachronous	16	11	5		16	5	11	
Peritoneal metastasis								
With	15	13	2	0.001	15	4	11	0.700
Without	14	3	11		14	5	9	
Primary site								
Colon	20	11	9	1.000	20	6	14	1.000
Rectum	9	5	4		9	3	6	
Unilateral / bilateral ovarian metastasis								
Unilateral	10	1	9	0.001	10	4	6	0.675
Bilateral	19	15	4		19	5	14	

The positive expression rate of EGFR in ovarian metastases was 65.5% (19/29). There was no significant difference in EGFR expression between ovarian metastases and the clinicopathological features (P > 0.05, Table 4).

Ten of 29 ovarian metastases were both EGFR and HER2 positive, 10′ cases were EGFR positive only, and 3 cases were HER2 positive only. The remaining 6 cases were negative for both EGFR and HER2. The correlation coefficient between EGFR and HER2 expression was r = 0.155 (*P* = 0.422), indicating that HER2 and EGFR expression in ovarian metastases was independent of one another.

In paired primary tumours and ovarian metastases samples, 5 out of 20 cases were HER2 positive in both primary tumours and ovarian metastases, 4 cases were HER2 positive in only primary tumours, and 5 cases were only positive in ovarian metastases, 9 cases were EGFR positive in both primary tumours and ovarian metastases, 5 cases were EGFR positive in only primary tumours, and 3 cases were only positive in ovarian metastases. The EGFR-positive rate was 69.0% in ovarian metastases, which was concordant with the 63.6% observed in the matched primary tumours (Tables 1 and 4).

### Relationship between HER2 expression and the prognosis of patients with CRC and ovarian metastases

**Fig. 2:**
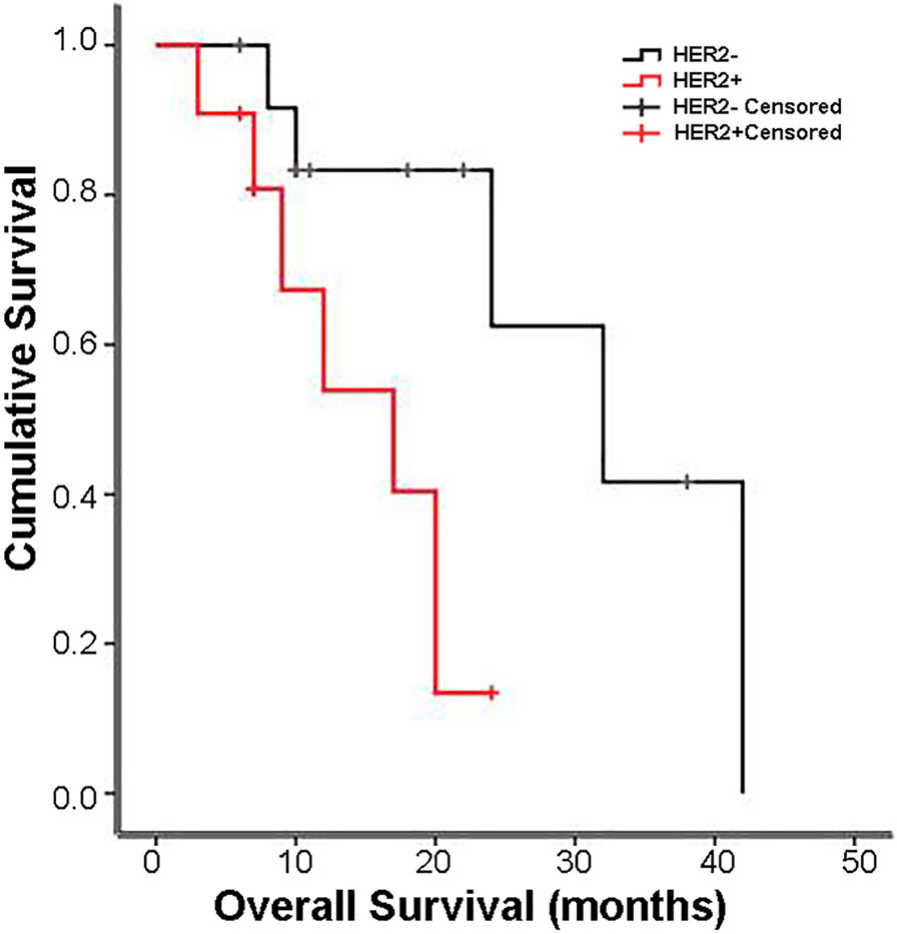
Kaplan-Meier survival curves of CRC patients with ovarian metastases as stratified by HER2 expression (*n* = 24)

Among all patients with ovarian metastases, the median survival time in metastases HER2-negative patients was 32.0 ± 8.3 months [CI 95%: 15.80–48.19], compared to 17.0 ± 5.2 months [CI 95%:6.76–27.24] in HER2-positive patients (Fig. 2).

## Discussion

EGFR and HER2 are all reported to be involved in the development and progress of CRC [8–11]. But their role in CRC ovarian metastases have not been clarified. In this respect, we analysed their expression and investigated their clinical importance in CRC patients with ovarian metastases.

Diverse rates of HER2 overexpression have been reported in CRC, with rates of membrane HER2 expression ranging from 2 to 11% due to a number of factors, including analysis of distinct subgroups of patients with heterogeneous clinicopathological features, use of different antibodies for IHC and diverse scoring systems (see review by Siena et al. [12]). More recent studies consistently indicated that HER2 overexpression accounts for approximately 2% of all CRCs [14, 15] and 7%~ 11% of stage III or IV tumours [14, 16, 17] are HER2 positive. Shan et al. reported that the HER2 positive rate in female CRC patients was 21.4% (6/26) vs. 8.4% (5/59) in males [17]. In our study, the HER2 positivity in primary tumours was 36.4% (8/22) in metastatic CRC patients with non-ovarian metastases. The observed differences may be caused by the stricter criteria they followed [18]. To date, very few cases with ovarian metastasis have been included in previous studies. In patients with ovarian metastases, the HER2 positive rate was even higher (Table 2), as 12 of 22 primary tumours (54.5%) and 13 of 29 ovarian metastases were HER2 positive in our study.

Recent studies showed that distal carcinomas were more likely to be HER2 or EGFR positive than proximal carcinomas despite the tests used [18, 19]. Higher frequencies of HER2 overexpression were found in rectal cancer than in descending colon or right colon cancers [15]. The results of our research showed that in patients with non-ovarian metastases, the frequency of HER2 overexpression in rectal cancer was higher than colon cancer though the differences was not statically significant due to small sample size. However, in patients with ovarian metastases, the frequency in rectal cancer and colon cancers were similar (Table 2). Among all ovarian metastases, a higher frequency of HER2 positivity in ovarian metastases was correlated with peritoneal metastasis and bilateral ovarian metastasis (*P* < 0.05) but not with age, synchronous or metachronous ovarian metastases or primary tumour location (*P* > 0.05) (Table 4).

Previous studies showed inconsistent results regarding the prognostic role of HER2 in CRC [14, 20–22]. Earlier studies proposed that HER2 overexpression was a negative prognostic factor [20, 21], but a recent trial concluded that HER2 amplification was not associated with patient outcomes [14, 22]. Nevertheless, for female patients with ovarian metastasis, HER2 overexpression in ovarian metastases indicated a worse overall survival time than that in HER2-negative patients (Fig. 2). However, whether HER2 overexpression could predict a higher risk of ovarian metastasis remains unknown. In addition, HER2 was reported to be a negative biomarker for EGFR-targeted treatments such as cetuximab and panitumumab in CRC [6, 23]. Thus, HER2 status should be considered when developing treatment strategies for those patients.

The role of EGFR was an important player in initiation and progression in CRC. EGFR overexpression was detected in 80–90% of colorectal tumours [8]. In the ERBITUX-OUEST study, 194 (58%) of metastatic CRC cases were EGFR positive [24]. Another study showed that 65% of the primary CRC tumours, 66% of the metastases, and 43% of the matched primary CRC metastases were EGFR positive [25]. In our study, the positive rates of EGFR were 63.6 and 58.3% in primary tumours of patients with ovarian and non-ovarian metastases, respectively (Table 1), which was in line with previous studies. Unlike HER2 positivity, EGFR positivity was not significantly correlated with clinicopathological features in CRC patients with ovarian metastases (*P* > 0.05).

## Conclusions

In conclusion, our studies revealed that HER2 and EGFR are highly expressed in ovarian metastases and primary tumours of CRC patients with ovarian metastases. In addition, HER2 positivity could be a negative prognosis predictor in patients with ovarian metastases.

## Abbreviations

CA125: Cancer Antigen 125
CDX2: Caudal Type Homeobox 2
CEA: carcinoembryonic antigen
CK20: Cytokeratin 20
CK7: Cytokeratin 7
CRC: colorectal cancer
EGFR: Epidermal growth factor receptor
HER2 (ERBB2, neu): Erb-b2 receptor tyrosine kinase 2
HER3 (ERBB3): Erb-b2 receptor tyrosine kinase 3
HER4 (ERBB4): Erb-b2 receptor tyrosine kinase 4
IHC: immunohistochemistry
PAX8: Paired box 8
PBS: phosphate buffered saline
